# Delayed Presentation of Air Embolism Within Cerebral Arteries Following Computed Tomography-Guided Lung Biopsy

**DOI:** 10.7759/cureus.33205

**Published:** 2023-01-01

**Authors:** Cunli Yang, Shao J Ong, Stanley E Loh, Gopinathan Anil

**Affiliations:** 1 Radiology, National University Hospital, Singapore, SGP; 2 Radiology, National University of Singapore, Singapore, SGP

**Keywords:** hyperbaric oxygen therapy (hbot), ct guided biopsy, thoracic radiology, metastatic cancer of unknown primary, iatrogenic complication, interventional radiology, acute ischaemic stroke, vascular air embolism, lung biopsy

## Abstract

Computed tomography (CT)-guided percutaneous core needle biopsy of the lung is a frequently performed interventional radiological procedure. Most complications are minor and self-resolving. However, a rare but potentially fatal complication is that of systemic air embolism, especially when to the cerebral or coronary arteries. This study reports a case of delayed (12 hours after initial biopsy) air embolism in the cerebral arteries that resulted from an otherwise uncomplicated biopsy of a lung nodule. It is vital for early diagnostic confirmation and appropriate treatment if possible, though maximal efforts at prevention are still recommended.

## Introduction

Percutaneous computed tomography (CT)-guided lung biopsy is a commonly performed procedure by interventional radiologists. The two most common complications from lung biopsies are pneumothorax and pulmonary haemorrhage. Air embolism is a rare complication with the potential to cause significant morbidity and mortality [1]. Ischaemic stroke secondary to air embolism from lung biopsy usually presents as immediate/early peri-operative complication seen within the initial few hours [2,3]. We report an unusually delayed (12 hours) presentation of post-lung biopsy air embolism leading to major acute ischaemic stroke.

## Case presentation

**Figure 1:**
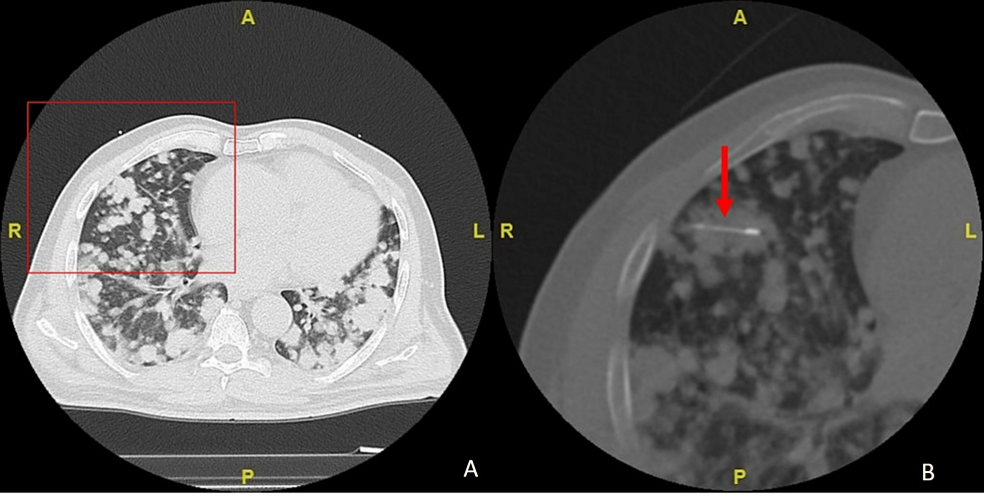
Axial Computed Tomography (CT) in lung window demonstrating multiple lung nodules (A). Magnified local view (B) of the segment framed red in (A) for accurate needle placement with a 15mm core biopsy needle (red arrow) within one of the largest cluster of nodules located peripherally in the right lower lobe.

A 65-year-old male with incidentally detected multiple lung nodule was referred for CT-guided lung biopsy of the nodules. The biopsy was performed by a consultant interventional radiologist, under local anaesthesia (1% lignocaine) with the patient in supine position. Under CT fluroscopy-guidance, a 17G guiding needle was introduced into one of the lung nodules in the right lower lobe. Using a coaxial technique, with an 18 G Trucut biopsy needle (AccuCore Coaxial Introducer needle and biopsy instrument; Inrad; Grand Rapids, MI, USA) with a 1.5cm throw. Three passes were made with three cores of tissue obtained. The target lesion was relatively peripheral in location and distant from any interlobular fissure, visible large airway or pulmonary vessel (Figure 1).

Following advancement into the target, the internal stylet of the guiding needle was timed for exchange for the core biopsy needle (and vice versa) whilst the patient was observed to begin respiratory expiration by his chest movements. A finger was used to occlude the co-axial introducer port during all exchanges to prevent air from being inadvertently sucked in. During the entire procedure, the patient’s heart rate, blood pressure and blood oxygenation levels were continuously monitored with no untoward change recorded. A post-procedure CT scan of the whole thorax was performed which excluded any procedural complication except for mild intraparenchymal haemorrhage around the biopsy tract. There was no pneumothorax, hemothorax or systemic gas locules in the visualised vessels or heart. Chest radiographs at one and six hours post-biopsy did not demonstrate any pneumothorax or effusion. The patient was discharged well after eight hours of close monitoring in a day-surgery ward. There was no documented incident of severe or persistent forced coughing during or after the procedure prior to discharge. 

**Figure 2:**
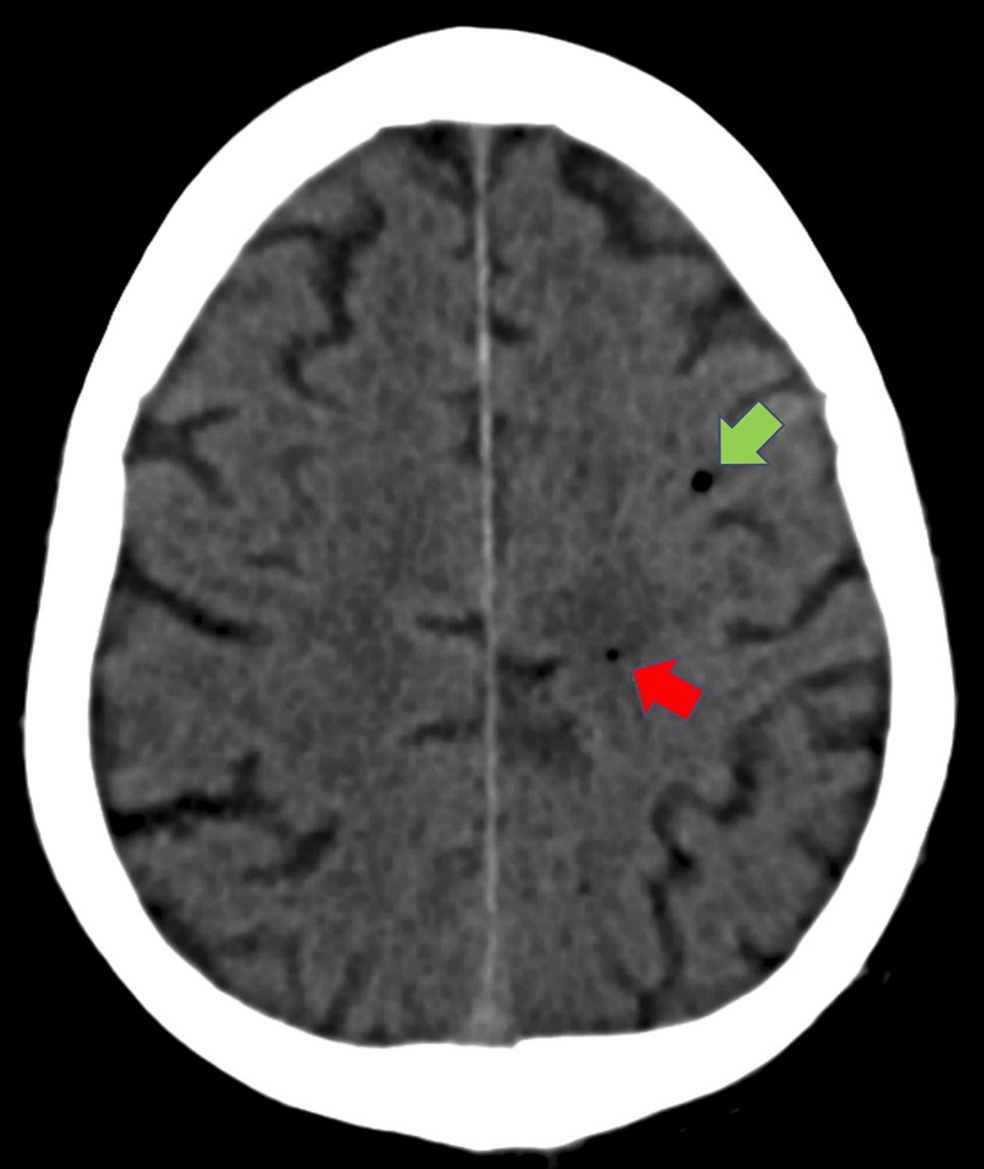
Unenhanced axial computed tomography (CT) brain demonstrating a few isolated air locules (green and red arrows) predominantly around the left anterior cerebral artery (green) – middle cerebral artery (red) watershed region.

Approximately 12 hours after the procedure, the patient developed sudden onset left-sided weakness. He soon became unresponsive and was admitted to his local hospital. An emergent CT examination of his head performed on admission as part of his stroke screen demonstrated tiny locules of gas in the subarachnoid vessels along the left cerebral convexity, predominantly around the left anterior cerebral artery-middle cerebral artery (ACA-MCA) watershed region (Figure 2).

**Figure 3:**
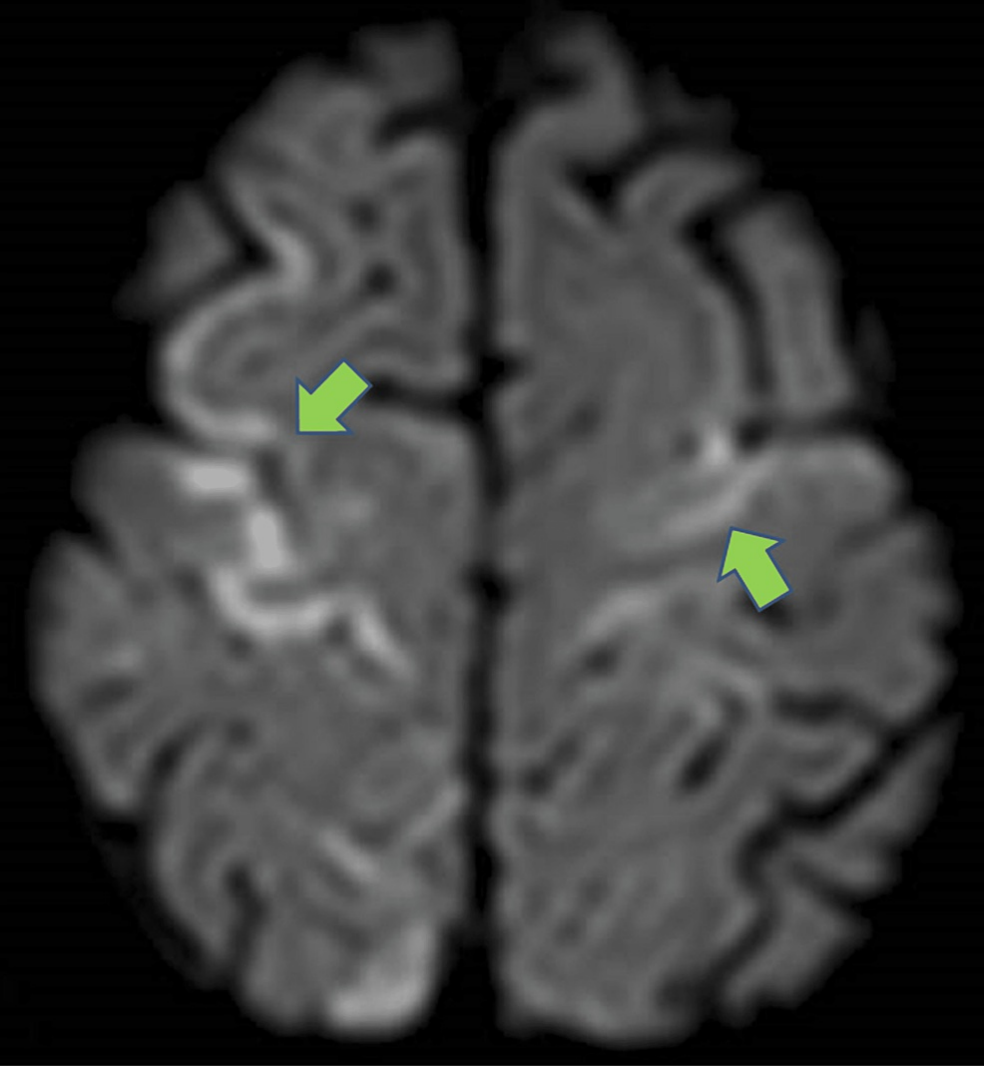
Magnetic resonance imaging (MRI) brain diffusion-weighted imaging (DWI) sequence showing gyriform foci of restricted diffusion in keeping with acute watershed infarcts as a result of earlier cerebral air emboli.

A magnetic resonance imaging (MRI) brain performed the next morning showed gyriform cortical/subcortical diffusion restriction in the bilateral fronto-parietal and right occipital lobes, along the arterial watershed areas; some of these corresponded to the distribution of aforementioned gas locules (Figure 3). 

A lumbar puncture was performed and cerebral spinal fluid (CSF) analysis was unremarkable. A contrast-enhanced two-dimensional echocardiography (2DE) excluded a right-to-left shunt. The patient was intubated and started on supportive measures. Repeat CT and MRI brain after a further 48 hours revealed spontaneous resolution of the air in the vessels with expected temporal evolution of the previously noted watershed infarcts.

The working diagnosis was acute infarcts secondary to air embolism and was managed accordingly. The lung biopsy results were in keeping with metastatic adenocarcinoma. One week after the incident, in view of the overall poor prognosis, the family decided to take the patient home for domiciliary palliative care.

## Discussion

The common complications of lung biopsies include pneumothorax (25.3%, with a smaller number [5.6%] requiring chest drain insertion), pulmonary haemorrhage (18.0%), and hemoptysis (4.1%). The risk of needle track seeding is 0.012-0.061%, and death is 0.16% [4]. In several large registries, air embolism as a complication of percutaneous lung biopsy is rare (0.02% to 0.07%) [1]. However, single-centre observational studies have reported higher air embolism rates of incidence of up to 3.8%, with 0.49% being symptomatic [5,6].

For air to enter the systemic circulation, a positive pressure gradient from the airway/air to pulmonary vein is required. Some of the possible mechanisms that can lead to a percutaneous lung biopsy-related systemic air embolism are 1) direct entry of air through a hollow needle open to the atmosphere with its tip in the pulmonary vein, 2) air introduced into the pulmonary artery may reach the pulmonary vein via traversing the pulmonary microvasculature, even in the absence of arteriovenous malformation, 3) post-biopsy fistulous communication between an aerated compartment of the lung and pulmonary vein with air being forced into the low-pressure pulmonary circulation during forced elevation of intrathoracic pressure for eg during spasmodic coughing. Other causes of systemic cerebral air embolization have also been reported in cases with haemodialysis circuits as well as endoscopy and central venous line insertions [7]. 

In this patient, the 12-hour interval between the presentation and biopsy as well as no evidence of intravascular air in the post-biopsy CT scan excludes the first possibility. To the best of our knowledge, there is no other reported instance of such a delayed presentation of post-lung biopsy air embolism. In previous reports, air embolism was either seen as an incidental finding and if symptomatic, would manifest in the early post-biopsy period, usually within minutes [2-6,8]. The most delayed presentation reported was a left hemispheric syndrome presenting several hours after biopsy. However, unlike our case, a small locule of gas was already present in the left atrium in retrospective review of the immediate post-biopsy CT thorax [6]. Hence, in our case, we hypothesize that the biopsy could have led to a small airway-venous fistula. This may have been initially sealed by the post-biopsy hemorrhage/atlectasis. We postulate that the tamponading effect may have petered out after the first few hours of biopsy, opening up an abnormal communication leading to the systemic air embolism. In addition, the biopsy target was in the right lower lobe adjacent to the right hemidiaphragm. Greater respiratory motion of the lung in this area, increased vessel calibre in the lower lobe as well as adjustments and redirections of needle in order to engage the target lesion could also have resulted in an increased possibility of a fistulous track/air embolism.

Even a small amount of air in the cerebral or coronary vessels can cause serious adverse effects. The former can present as focal neurological deficits, seizures and/or altered sensorium while the latter may cause chest pain, hypotension and cardiac arrhythmias. Fatal consequences with injections of as less as 2ml of air into the cerebral circulation and 1ml into the coronary arteries are known [8]. A high index of suspicion is essential to identify this complication in a timely manner and manage it appropriately.

Upon any suspicion for air embolism, 100% O2 administration should be started with the patient preferably placed in the right lateral decubitus position (right side down, such that the left ventricular outflow tract lies inferiorly) to reduce the chance of air entering the systemic circulation [9]. The emergency response team should be activated, and CPR administered if necessary. Once the patient has stabilised, further imaging of the whole thorax as well as the brain can be performed to identify systemic air embolization. The definitive treatment of cerebral air embolism is hyperbaric oxygen (HBOT) [10]. It provides 100% oxygen at high pressure. The high pressure compresses the air bubbles, thereby reducing their size, relieving obstruction. The high partial pressure of 100% oxygen would help dissolve the nitrogen within the residual bubbles with the aim of improving tissue oxygenation. It also decreases intracranial pressure, decreases reperfusion injury, and reduces mortality among severely brain-injured patients [11]. Generally, prognosis is good if HBOT is started within six h of the insult. Delays caused by other priorities should however not discourage the use of HBOT. In fact, successful treatment of air embolism possibly up to 40 h after initial onset of neurologic deficit has been detailed [10].

The risk of post-biopsy air embolism can be reduced by avoiding a prone position during biopsy [5]. Supine or lateral decubitus positioning is preferred, as air is more likely to be trapped in a pocket within the left heart before it gets further into the systemic circulation. Some authors suggest instructing the patient to suspend respiration and not cough during the biopsy procedure [6]. However, in our experience, on most occasions these are impractical. Attempts at breath-holding may often be followed by unexpectedly deep inspiratory efforts or even bouts of coughing. It is preferable to allow the patient to continue with normal quiet breathing. As a precaution, we close the hollow guiding needle of a coaxial biopsy set with a finger/stylet when the biopsy needle is not within it. It is also good practice to carefully review the post-biopsy CT scan for any evidence of intravascular/intracardiac air before it manifests clinically [6].

## Conclusions

Cerebral air embolism after percutaneous lung biopsy is a rare but potentially fatal complication. Prompt recognition and management are crucial to optimise outcomes. With most lung biopsies progressively being performed as outpatient or day-surgery procedures with short hours of post-biopsy monitoring due to resource limitations, it is imperative for both patients and caregivers to be aware and cognizant of the risk and signs and symptoms of these delayed complications.
